# Characterization of the angular gyrus in an older adult population: a multimodal multilevel approach

**DOI:** 10.1007/s00429-022-02529-3

**Published:** 2022-07-29

**Authors:** Christiane Jockwitz, Camilla Krämer, Johanna Stumme, Paulo Dellani, Susanne Moebus, Nora Bittner, Svenja Caspers

**Affiliations:** 1 Institute of Neuroscience and Medicine (INM-1), Research Centre Jülich, Jülich, Germany; 2 Institute for Anatomy I, Medical Faculty & University Hospital Düsseldorf, Heinrich Heine University, Düsseldorf, Germany; 3 Institute of Urban Public Health, University of Duisburg-Essen, Essen, Germany

**Keywords:** Angular gyrus, Aging, Brain structure, Resting-state functional connectivity, Structural connectivity, Cognition, Lifestyle

## Abstract

The angular gyrus (AG) has been associated with multiple cognitive functions, such as language, spatial and memory functions. Since the AG is thought to be a cross-modal hub region suffering from significant age-related structural atrophy, it may also play a key role in age-related cognitive decline. However, the exact relation between structural atrophy of the AG and cognitive decline in older adults is not fully understood, which may be related to two aspects: First, the AG is cytoarchitectonically divided into two areas, PGa and PGp, potentially sub-serving different cognitive functions. Second, the older adult population is characterized by high between-subjects variability which requires targeting individual phenomena during the aging process. We therefore performed a multimodal (gray matter volume [GMV], resting-state functional connectivity [RSFC] and structural connectivity [SC]) characterization of AG subdivisions PGa and PGp in a large older adult population, together with relations to age, cognition and lifestyle on the group level. Afterwards, we switched the perspective to the individual, which is especially important when it comes to the assessment of individual patients. The AG can be considered a heterogeneous structure in of the older brain: we found the different AG parts to be associated with different patterns of whole-brain GMV associations as well as their associations with RSFC, and SC patterns. Similarly, differential effects of age, cognition and lifestyle on the GMV of AG subdivisions were observed. This suggests each region to be structurally and functionally differentially involved in the older adult’s brain network architecture, which was supported by differential molecular and genetic patterns, derived from the EBRAINS multilevel atlas framework. Importantly, individual profiles deviated considerably from the global conclusion drawn from the group study. Hence, general observations within the older adult population need to be carefully considered, when addressing individual conditions in clinical practice.

**Supplementary Information:**

The online version contains supplementary material available at 10.1007/s00429-022-02529-3.

## Introduction

The angular gyrus (AG) is a heterogeneous brain structure that has been associated with a variety of cognitive functions, including language functions (i.e., semantic information processing), spatial and memory functions, number and attentional processing, social cognition as well as multisensory perception (Binder et al. 2009; Seghier 2013; Humphreys et al. 2021). During the aging process, the AG has been shown to undergo substantial structural atrophy starting during midlife and continuing until older ages (Walhovd et al. 2005; Fjell et al. 2009, 2013; Jockwitz et al. 2017a). Furthermore, associations between gray matter volume (GMV) of the AG and subjective and mild cognitive impairment (MCI) as well as dementia have been reported (Yao et al. 2012; Quiroz et al. 2013; Oh et al. 2014; van de Mortel et al. 2021; Zhang et al. 2021). For example, Karas et al. (2008) showed that subjects who converted from MCI to Alzheimer’s disease showed higher atrophy in the left AG as compared to those who remained mild cognitively impaired. Likewise, subjects suffering from subjective cognitive impairment (Kim et al. 2019) were depicted by lower GMV within the AG, as well as lower structural network connectivity between the AG and superior parietal and pre- and postcentral gyri, which, in turn, was associated with their cognitive decline. Hirst et al. (2021) even highlighted the AG as possible cross-modal hub region associated with age-related changes in multisensory perception. Hence, previous results hint at a key role of the AG during the aging process with a potential relation to neurodegenerative diseases and cognitive decline.

By exploring the key role of the AG during the aging process, previous research often focused on the AG as a macro-anatomical entity. Commonly, it is anatomically summarized together with the rostrally lying supramarginal gyrus as the inferior parietal lobule (Seghier 2013), or integrated into functional units such as the temporoparietal junction including posterior parts of the temporal lobe (Schurz et al. 2017). Yet, these approaches disregard multimodal evidence that the AG is a composition of two micro-anatomically distinct structures. For instance, post-mortem cyto-architectonic analyses revealed the AG to be subdivided into a rostrally lying area PGa and a caudally lying area PGp, which were shown to be involved in distinct functional brain networks and hence related to different cognitive functions (Caspers et al. 2006, 2008). Based on this multimodal evidence, the examination of these AGs subdivisions may be highly promising to further unravel the potential key role of the AG in terms of age-related differences and its association to behavior.

To this aim, when focusing on the older adult population, we are confronted with a particularly high inter-individual variability at the level of behavior, brain structure as well as brain function (Habib et al. 2007; Dickie et al. 2013). Precisely, from early to late adulthood, the factor ‘age’ is able to explain large parts of the variance in terms of both, cognitive abilities and brain structural parameters (Hedden and Gabrieli 2004; Schaie 2009). Focusing on samples of only older adults, however, reveals a quite different picture. Here, the factor ‘age’ alone is not able to explain large parts of the variance. Despite the consideration of other factors, such as lifestyle (Bittner et al. 2019; Hamer and Batty 2019), sex (Jahanshad and Thompson 2017; Jockwitz et al. 2021a, b), genetic predispositions (Honea et al. 2009; Caspers et al. 2020), or environmental influences (de Prado Bert et al. 2018; Nussbaum et al. 2020; Lucht et al. 2022), the high inter-individual heterogeneity remains only partly explained. In fact, to identify general brain–phenotype relations in the older adult population on the group level, where each factor might show small effect sizes, very large sample sizes are required (Button et al. 2013). On the other hand, averaging behavior across large groups may suppress and underestimate differences on the individual level (Jockwitz et al. 2021a, b). Identifying global trends in these large samples with high inter-individual variability comes at the cost of losing perspective on and neglecting the specific pattern of influencing factors of individual subjects, as is particularly important in case of personalized treatment considerations. As these individual profiles might deviate considerably from the global conclusion drawn from the group study, individual deep phenotyping approaches are required to uncover the relevance of different factors for each individual to explain observed differences in the aging AG.

Consequently, the investigation of a possible key role of AG in the aging brain requires the consideration of two essential aspects: first, a functionally meaningful definition of AG sub-regions, as available through the Julich-Brain atlas (Amunts et al. 2020), (i.e., areas PGa and PGp) and second, a specific focus on the diverse multimodal profiles of factors influencing the aging process of the AG sub-regions in individuals. The current study first employed a multimodal, multilevel (i.e., brain structure, functional and structural connectivity) characterization of the AG subdivisions PGa and PGp in a large population-based study of older adults. Such multimodal investigations have been proven to be useful since different brain modalities were found to be distinctively related to differences in brain structure. For example, previous studies revealed that during the aging process, global as well as regional GMV decreases would be associated with both, positive and negative changes in brain connectivity of functional networks sub-serving cognitive functions in the older adult population (Jockwitz et al. 2017; Stumme et al. 2020; Spreng et al. 2016; Reuter-Lorenz et al. 2011). We here built upon this principle and examined the association between GMV of the AG subparts and either GMV, resting-state functional connectivity [RSFC] or structural connectivity [SC] of all ROIs included in the Julich-Brain Atlas to explore brain–brain relationships in a systemic approach. Furthermore, we additionally consulted the EBRAINS (https://ebrains.eu), a multilevel atlas framework, to characterize the ROIs on the molecular and gene expressions level. Second, group analyses were conducted to assess the relation between AG structure and age, cognition and lifestyle. Finally, we switched to the “individual view”. For this purpose, we selected individuals who exhibited either a particularly high or low GMV within the AG to subsequently highlight their respective individual cognitive and lifestyle profiles compared to the overall study sample.

## Methods

### Subjects

All subjects included in the current study were drawn from 1000BRAINS (Caspers et al. 2014), a population-based cohort study, recruited from the Heinz Nixdorf recall study that has been conducted in the Ruhr area in Germany (Schmermund et al. 2002). Exclusion from the study was based on eligibility for MR measurements for scientific purposes. From the initial cohort of 1314 subjects, we selected subjects being 55 years and older (*n* = 969).

Furthermore, subjects being at risk for dementia [as measured using the DemTect; (Kalbe et al. 2004)] were excluded (*n* = 31). From these 938 subjects with available data sets for cognitive performance, brain structure (available for *n* = 878 subjects), RSFC (available for *n* = 829), SC (available for *n* = 685) and lifestyle (available for *n* = 499) have been selected. All participants gave written informed consent before participating in 1000BRAINS. All experiments were performed in accordance with relevant guidelines and regulations. The study protocol was approved by the local Ethics Committee of the University of Essen.

### Cognitive performance and lifestyle

All subjects underwent intensive neuropsychological testing during their participation in 1000BRAINS. In total, 16 different cognitive functions, namely selective attention, processing speed, reasoning, concept shifting, susceptibility to interference, figural fluency, phonematic and semantic verbal fluency (with and without switching between different letters/semantic categories), vocabulary, verbal episodic memory, figural memory, visual, visual–spatial and verbal short-term (STM)/working memory (WM) were assessed. For detailed information, see (Caspers et al. 2014; Jockwitz et al. 2017). In terms of lifestyle behavior (Ainsworth et al. 2011, Bittner et al. 2019), we assessed information regarding alcohol consumption (yes-no), body mass index (BMI), dietary index (Frolich et al. 2017), smoking behavior (never-ever-current), social integration (social integration index) and sports (metabolic equivalent, Ainsworth et al. (2011)). For an overview of parameters used, mean values and standard deviations, see Table 1.

**Table 1:** Variables included in the current study with mean of raw values and corresponding standard deviations (SD) respectively the proportion *n* (%)

	Variable	Mean (SD)		Variable	Mean (SD)
Demographics	Age (years)	67.4 (6.6)	Cognition	Selective attention (time in sec.)	34.76 (11)
	Sex	1.46 (0.5)		Processing Speed (time in sec.)	40.52 (14.37)
	Education	6.41 (1.97)		Reasoning (correct answers)	20.37 (5.09)
	TBV	1473.55 (132.81)		Interference (time in sec.)	43.34 (22.68)
GMV of AG				Concept shifting (time in sec.)	55.41 (37.87)
	lPGa	1.84 (0.26)		Visual spatial STM (correct answers)	5.44 (0.88)
	rPGa	2.95 (0.38)		Visual spatial WM (correct answers)	4.66 (1.06)
	lPGp	4.31 (0.53)		Visual WM (correct answers)	7.65 (1.77)
	rPGp	3.81 (0.47)		Verbal STM (correct answers)	6.06 (1.07)
Lifestyle				Verbal WM (correct answers)	4.65 (1.07)
	Packyears	13.35 (21.78)		Figural fluency (correct answers)	26.02 (7.26)
	Dietary index	11.39 (18.34)		Phonematic fluency (correct answers)	18.71 (6.58)
	BMI	0.92 (0.1)		Semantic fluency (correct answers)	23.76 (6.83)
	Sports (metabolic equivalent)	37.69 (107.51)		Phonematic fluency switch (correct answers)	18.86 (6.09)
	Social integration index	11.97 (3.3)		Semantic fluency switch (correct answers)	19.87 (4.79)
	Alcohol consumption	Yes (*n* =202; 40.5%); No (*n* = 297; 59.5%)		Vocabulary (correct answers)	30.86 (4.9)
	Smoking	Never (*n* = 219; 43.9%); ever (*n* = 233; 46.7%); current (*n* = 41; 8.2%)		Figural memory (correct answers)	8.59 (4.12)
				Verbal memory (correct answers)	41.68 (10.29)

Total brain volume; *BMI* body mass index; *STM* short-term memory; *WM* working memory

### Image acquisition

All brain images were acquired in the frame of the imaging protocol of 1000BRAINS (Caspers et al. 2014) using a 3T Siemens Tim-TRIO MR scanner with a 32-channel head coil. For the purpose of the current study, the following sequences were of interest: (1) 3D high-resolution T1-weighted magnetization-prepared rapid acquisition gradient-echo (MPRAGE) (176 slices, slice thickness = 1, TR = 2250 ms, TE = 3.03 ms, FoV = 256 × 256 mm^2^, flip angle = 9°, voxel resolution = 1 mm^3^); (2) 300 gradient-echo planar (EPI) images (slices = 36, slice thickness = 3.1 mm, TR = 2200 ms, TE = 30 ms, FoV = 200 × 200 mm^2^, voxel resolution = 3.1 mm^3^; participants were instructed to keep their eyes closed, to relax and let their mind wander, but not to fall asleep, which was checked during a post-scan debriefing) and (3) diffusion-weighted images (DWI) with two different *b*-values: *b =* 1000 s/mm^2^ (HARDI subset, EPI, TR = 6.3 s, TE = 81 ms, 7 b0-images (interleaved) and 60 diffusion-weighted volumes, voxel resolution = 2.4 mm^3^) and *b* = 2700 s/mm^2^ (HARDI subset, EPI, TR = 8 s, TE = 112 ms, 13 b0-images (interleaved) and 120 diffusion-weighted volumes, voxel resolution = 2.4 mm^3^).

### Brain image analyses

#### Brain regions of interest

**Fig. 1:**
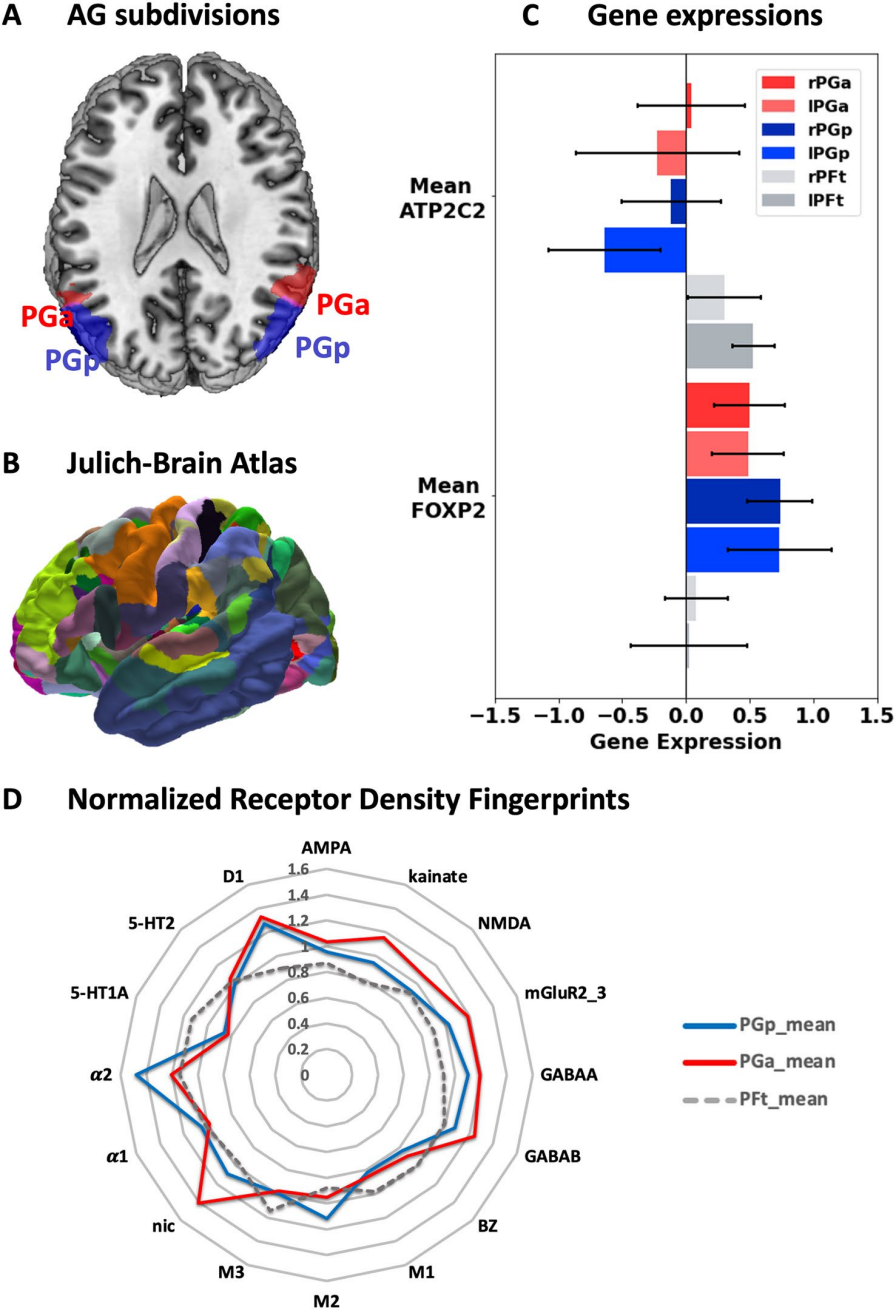
**A** AG subdivisions PGa and PGp; **B** 3D Visualization of the Julich-Brain Atlas; **C** normalized gene expressions of the two genes of interest: ATP2C2 and FOXP2. Areas PGa is colored in red (light red = left PGa), areas PGp are colored in blue (lighter blue =left PGp), areas PFt (part of supramarginal gyrus, colored in grey, light grey = right PFt) serves as control region; **D** normalized receptor densities fingerprints for 15 receptors as reported by Caspers et al. (2013). Area PGa is colored in red, area PGp is colored in blue, area PFt is colored in grey and serves as control region

For the purpose of the current study, the regions of interest included left and right PGa and PGp within the AG as defined by Caspers et al. 2006, 2008, 2013. Both areas, PGa and PGp are part of the cyto-architectonically defined Julich-Brain atlas (version 2.6; https://search.kg.ebrains.eu/instances/Dataset/2eaa3dc6-a21b-41c1-b703-bf06f82adf25 ; (Amunts et al. 2020). All other areas included in the Julich-Brain atlas served as regions for network analyses of AG alterations (for areas PGa and PGp, see Fig. 1A; for the Julich-Brain atlas as represented in EBRAINS, see Fig. 1B).

#### Brain structure

From the T1-weighted structural brain images, we extracted GMV (in ml) using the standalone version of the CAT12v8 SPM12 toolbox, release 1853 (Franke and Gaser 2019)) for all cyto-architectonically defined areas of the Julich-Brain atlas as well as the total brain volume (TBV). This included (a) initial registration and bias field correction, (b) segmentation into tissue probability maps (TPM) of gray matter, white matter, and cerebrospinal fluid, (c) a spatial normalization to the standard template derived from 555 healthy subjects between 20 and 80 years of the IXI-database (http://www.brain-development.org) computed using the geodesic shooting and Gauss–Newton optimisation-based diffeomorphic registration (Ashburner and Friston 2011).

#### Resting-state functional connectivity

For each participant, the first four echo planar imaging (EPI) volumes were discarded. Using a two-pass procedure, all functional images were corrected for head movement by fist aligning all volumes to the first image and second to the mean image using affine registration. By the use of the “unified segmentation” approach (Ashburner and Friston 2005), all functional images were spatially normalized to the MNI152 template (Holmes et al. 1998; Calhoun et al. 2017; Dohmatob et al. 2018). Additionally, ICA-based Automatic Removal Of Motion Artifacts [ICA-AROMA (Pruim et al. 2015)] was applied to identify and remove motion-related independent components from functional MRI data. Afterward, global signal regression was applied to minimize the relationship between motion and RSFC (Burgess et al. 2016; Ciric et al. 2017; Parkes et al. 2018). Lastly, all RS-fMRI images were bandpass-filtered (0.01–0.1 Hz). For RSFC, a mean time series were extracted for each region of interest using fslmeants (Smith et al. 2004) and correlated with the mean time series of the AG parts (left and right PGa and PGp) using Pearson’s correlations.

#### Structural connectivity

First, DWI data were corrected for eddy current and motion artifacts including interpolation of slices with signal dropouts (Andersson and Sotiropoulos 2015; Andersson et al. 2016). Suboptimal volumes or datasets (ghosting, remaining signal dropouts or very noisy data) were removed from further analysis. Afterwards, brain masks were created, all DWI data were rigidly aligned to the T1-weighted data set using mutual information as cost function (Wells et al. 1996) and resampled to 1.25mm. B-vectors were rotated according to the transformations.

Regarding distortion correction Anisotropic Power Maps (APM; Dell’Acqua et al. 2014) were computed from the b2700 DWI data in 1.25 mm space, which were used to compute the non-linear transformation from diffusion to anatomical T1 space taking EPI induced distortions into account. These non-linear transformations were used to transform the TPMs to diffusion space for computing an optimally fitting brain mask for the DWI data in the absence of field maps and b0 volumes with opposite EPI readout directions. All transformation steps were computed using the Advanced Normalization Tools (ANTs) version 2.1.1 (Avants et al. 2014).

The two datasets with b1000 and b2700 were merged into one single file and corrected for different echo times. This correction was computed by voxel-wise multiplying the b2700 data with the ratio of the non-diffusion-weighted data of the two datasets.

##### Calculation of CSD and streamlines

Local modeling and probabilistic streamline tractography were performed using the MRtrix software package (Tournier et al. 2012), version 0.3.15 (https://www.mrtrix.org/). The constrained spherical deconvolution (CSD) local model was computed using multi-tissue CSD of multi-shell data (Jeurissen et al. 2014) using all shells and a maximal spherical harmonic order of 8. Ten million streamlines were computed with dynamic seeding in the gray-white matter interface for every subject using the probabilistic iFOD2 algorithm and the anatomically constrained tractography framework (Smith et al. 2012) with a maximal length of 250 mm and a cut-off value at 0.06. Afterwards, optimized Spherical-deconvolution Informed Filtering of Tractograms (SIFT2) was applied to match the whole-brain tractograms to the fixel-wise fiber densities (Smith et al. 2015).

##### Connectivity matrices

Next, both maps were merged into one single file, dilated using fslmaths and transformed into diffusion native space using the SyN algorithm (ANTs 2.1.1). The whole-brain atlas in diffusion native space, the whole-brain tractogram and the SIFT2 weights per streamline were then fed into tck2connectome (MRtrix 0.3.15). This resulted in a symmetric 248 × 248 matrix which contained the sum of streamline weights per ROI combination per subject.

### Statistical analyses based on the total sample

In the first part, we aimed at characterizing the AG subparts in terms of brain structure, functional and structural connectivity. Therefore, GMV of the AG subdivisions were related to either region-wise whole-brain GMV, RSFC or SC (in parts of the Julich-Brain atlas). All three analyses were carried out using multiple regression analyses (forward-selection approach with sex, education and TBV as additional predictors) with GMV of the respective AG subdivision as independent variable and (a) GMV, (b) RSFC between AG subdivision and all other parts for the Julich-Brain atlas and (c) SC between AG subdivision and all other parts for the Julich-Brain atlas as dependent variables.

Second, to assess brain–phenotype relations for the two subdivisions of the left and right AG (areas PGa and PGp), we calculated several multiple regression analyses: First, we assessed the influence of age on GMV of the AG subparts using the inclusion approach (with sex, education and TBV as additional predictors). For behavioral variables, we included either cognitive performance test scores or lifestyle variables as explanatory variables using forward-selection approaches (with age, sex, education and TBV as additional predictors).

To target individual subject assessments, we selected five subjects with low GMV and five subjects with high GMV (participants being within the highest or lowest 25% regarding GMV for all four parts of the AG). For these ten subjects, we created individual profiles regarding cognitive performance and lifestyle (visualized as bar plots). To do so, we calculated standard Z-scores to create comparable scores for all variables.

### Consultation of multilevel atlas framework EBRAINS

In the frame of characterizing the AG subdivisions, we consulted regional genetic and molecular data using EBRAINS (https://ebrains.eu), a multilevel atlas framework, to explain the involvement of the AG subdivisions with respect to general as well as individual phenomena of the aging process. In terms of genetic data, we explored the JuGex tool (Bludau et al. 2018; https://ebrains.eu/service/jugex/). Since previous research claimed the involvement of the AG in language processing, we focused on genetic expression of language-related genes, i.e., FOXP2 and ATP2C2 (Lai et al. 2003; Newbury and Monaco 2010; Lambert et al. 2011; Unger et al. 2021a, b). While FOXP2 is supposed to be involved in the development of speech and language, ATP2C2 has been associated with dyslexia and other communication disorders (Lai et al. 2003; Newbury and Monaco 2010; Lambert et al. 2011; Unger et al. 2021a, b).

Functional differences in the individual regions were additionally investigated with respect to their receptor density fingerprints, since the function of a cortical area requires a well-matched receptor balance. We thus focused on the receptor density profiles of the AG subdivisions, as examined by Caspers et al. (2013) with densities of 15 different receptors (glutamatergic (α-amino-3-hydroxy-5-methyl-4-isoxazolepropionic acid [AMPA], kainate, *N*-methyl-d-aspartate [NMDA]), γ-aminobutyric acid (GABA)ergic (GABAA-, GABAB-, GABAA-associated benzodiazepine-binding sites), cholinergic (nicotinic, muscarinic M1, M2, M3), adrenergic (α1, α2), serotoninergic (5-HT1A, 5-HT2), and dopaminergic (D1)) which were measured in areas PGa and PGp in postmortem brains. We normalized the receptor densities (measured in fmol/mg) across all brain regions within the inferior parietal lobule (areas supramarginal gyrus: PF, PFt, PFop, PFm, PFcm; AG: PGa and PGp) by calculating the mean density values for each receptor and dividing the region-specific density value by the mean value. Finally, we compared the normalized receptor densities between areas PGa and PGp and additionally used area PFt as a control region.

## Results

We performed a multimodal characterization of the AG subdivisions including data regarding GMV, RSFC and SC together with genetic and molecular information. Using demographics, cognition and lifestyle, we then identified brain–behavior relationships at the group level. Finally, we identified individual profiles for subjects showing low and high GMV in areas PGa and PGp (see Fig. 1A for brain regions of interest).

### Group-derived relationships between GMV of the AG and its brain integration

**Fig. 2:**
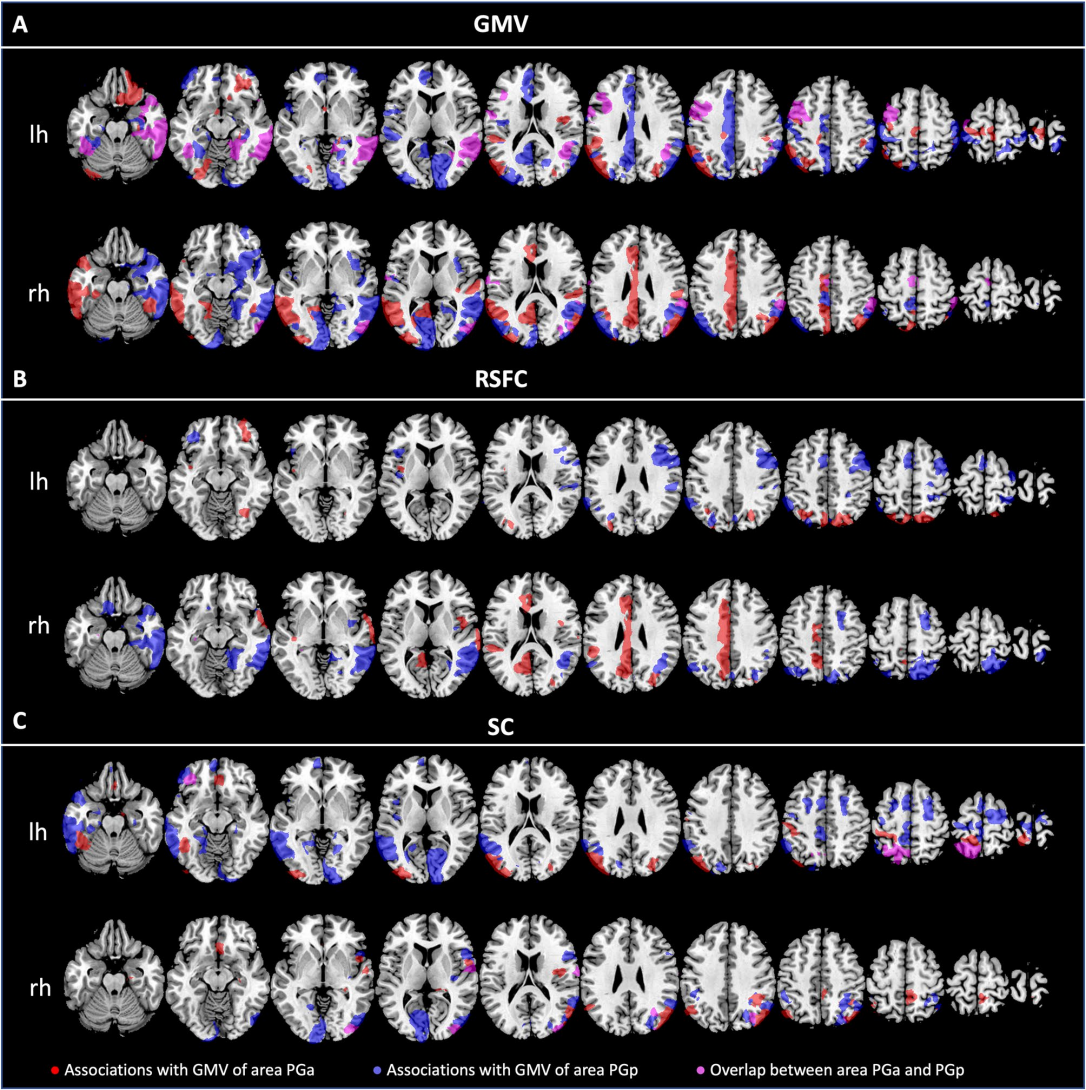
Brain regions being significantly associated with GMV of the AG subdivisions (areas PGa and PGp) in terms of **A** GMV; **B** RSFC and **C** SC (irrespective of the direction of the effects). All associations with GMV of area PGa are colored in red, while associations with GMV of area PGp are colored in blue. Associations with both areas PGa and PGp are colored in pink. Results are shown for the left and right hemispheric AG subdivisions separately. *lh* left hemisphere, *rh* right hemisphere

For associations between GMV and brain metrics, again, several different forward-selection multiple regression analyses were performed. We first addressed the relationship between GMV of the AG subdivision as dependent variables and GMV of all regions included in the Julich-Brain atlas. Figure 2A shows those brain regions significantly related to GMV of the AG subdivisions.

**Fig. 3:**
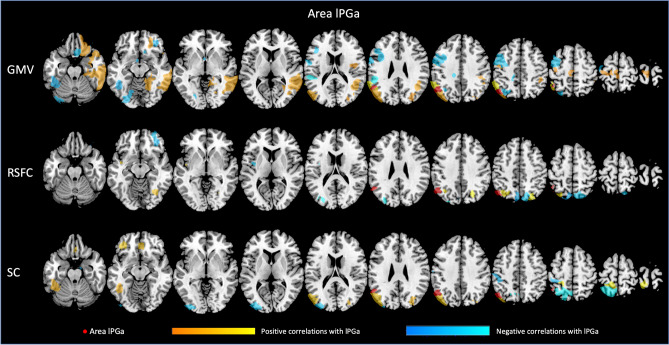
Significant associations between GMV of left area (l) PGa and GMV, RSFC and SC of those areas included in the Julich-Brain atlas

**Fig. 4:**
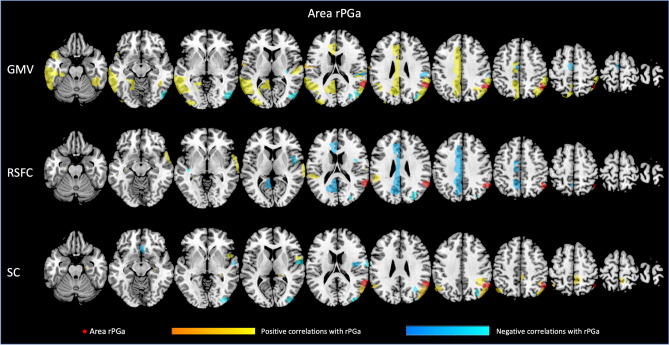
Significant associations between GMV of right area (r) PGa and GMV, RSFC and SC of those areas included in the Julich-Brain atlas

**Fig. 5:**
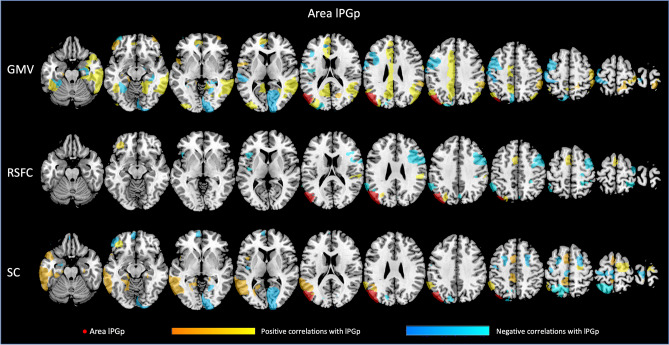
Significant associations between GMV of left area (l) PGp and GMV, RSFC and SC of those areas included in the Julich-Brain atlas

**Fig. 6:**
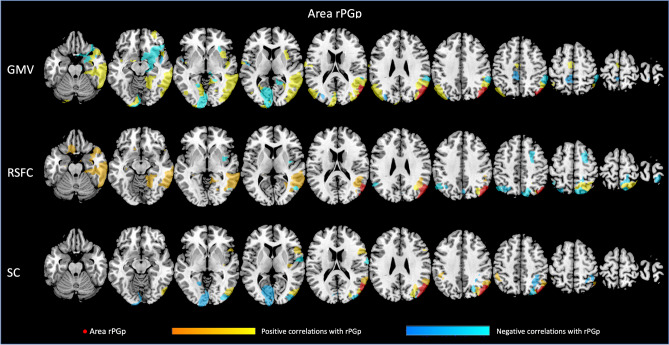
Significant associations between GMV of right area (r) PGp and GMV, RSFC and SC of those areas included in the Julich-Brain atlas

Each of the four regions investigated seems to be related to GMV of different brain regions, with both, positive and negative associations. For all associations between GMV of the AG subdivisions and other parts of the Julich-Brain atlas, please refer to Figs. 3, 4, 5, 6 as well as Suppl. Table S1 (for exemplary scatterplots, see Figure S5), and for the comparison of associations between left and right areas PGa and PGp refer to Fig. 2A–C. Exemplarily, GMV of left PGa was associated with GMV of other sub-regions of the left inferior parietal lobule, as well as with the left dorsolateral prefrontal and orbitofrontal cortex, while GMV of right PGa was associated with GMV of other sub-regions of the right inferior parietal lobule. Moreover, GMV of left and right area PGp was additionally negatively associated with areas of the respective contralateral primary visual cortex.

In a next step, we addressed the association between GMV of AG regions and RSFC between AG and all other parts of the Julich-Brain Atlas (Fig. 2B; for positive and negative associations, see Figs. 3, 4, 5, 6 and Table S2, for exemplary scatterplots, see Figure S5). In general, GMV of the left PGa was associated with RSFC between left PGa and left inferior parietal lobule and the intraparietal sulcus. GMV of the left PGp was associated with RSFC between left PGp and left inferior parietal lobule as well as the right dorsolateral prefrontal cortex. GMV of the right PGa was associated with RSFC between right PGa and left cingulate cortex, while GMV of right PGp showed widespread RSFC associations with parts of the left and right inferior parietal lobule, as well as parts of the temporal lobe.

Likewise, we addressed the association between GMV of AG regions and SC between AG and all other parts of the Julich-Brain Atlas (Fig. 2C; for positive and negative associations, see Figs. 3, 4, 5, 6 and Table S3, for exemplary scatterplots, see Figure S5). Here, again, each AG subdivision revealed a distinct composition of SC patterns related to the GMV of the AG subdivisions. GMV of left PGa was associated with SC between left PGa and left inferior and superior parietal lobule and intraparietal sulcus. GMV of left PGp was associated with SC between PGp and left parietal lobule, and with the primary visual cortex. GMV of right PGa was associated with SC between right PGa and right inferior and superior parietal lobule and intraparietal sulcus, with additional connections to right dorsal premotor cortex. In turn, GMV of right PGp was associated with SC between right PGp and, right inferior parietal lobule and intraparietal sulcus, and with the primary visual cortex and lateral occipital cortex.

Finally, we were interested whether the distinct involvement of the AG subdivisions in brain metrics (i.e., GMV, RSFC, SC) would be reflected in its regional genetic and molecular architecture. This was assessed by integrating information from the EBRAINS multilevel atlas framework. Regarding the molecular composition of the inferior parietal lobule, areas PGa and PGp show regional differences in receptor density fingerprints. Figure 1D represents the normalized receptor density fingerprints of areas PGa and PGp as well as area PFt, as one exemplary control region within the supramarginal gyrus. In general, the normalized receptor fingerprints of the two AG regions have a similar shape, whereas area PFt within the supramarginal gyrus is clearly differentiated from these two. For instance, area PFt showed a substantially lower receptor density of the D1 receptor, but also of the GABA_A_ receptor. Comparing the two AG regions, area PGp is characterized by high concentrations of the αlpha2 receptor as compared to area PGa, whereas area PGa shows exceptionally high concentrations of the nicotinic receptor compared to area PGp.

Since the AG is involved in language functions, we were additionally interested in whether this also manifests in distinct gene expressions. To do so, we additionally examined several gene expressions of language-related genes, using the JuGex tool in EBRAINS (Bludau et al. 2018). Figure 1C represents the normalized gene expressions for the AG parts as well as supra-marginal area PFt. Regarding ATP2C2, we found lower gene expressions within the two AG parts as compared to area PFt, while for FOXP2, we found opposite patterns, i.e., higher gene expressions for areas PGa and PGp as compared to area PFt. Comparing the two AG sub-regions, we found lower gene expressions for FOXP2 in area PGp compared to area PGa bilaterally and higher gene expressions of ATP2C2 in area PGp compared to PGa.

### Group analyses of AG subdivisions in light of age, cognitive performance and lifestyle

**Fig. 7:**
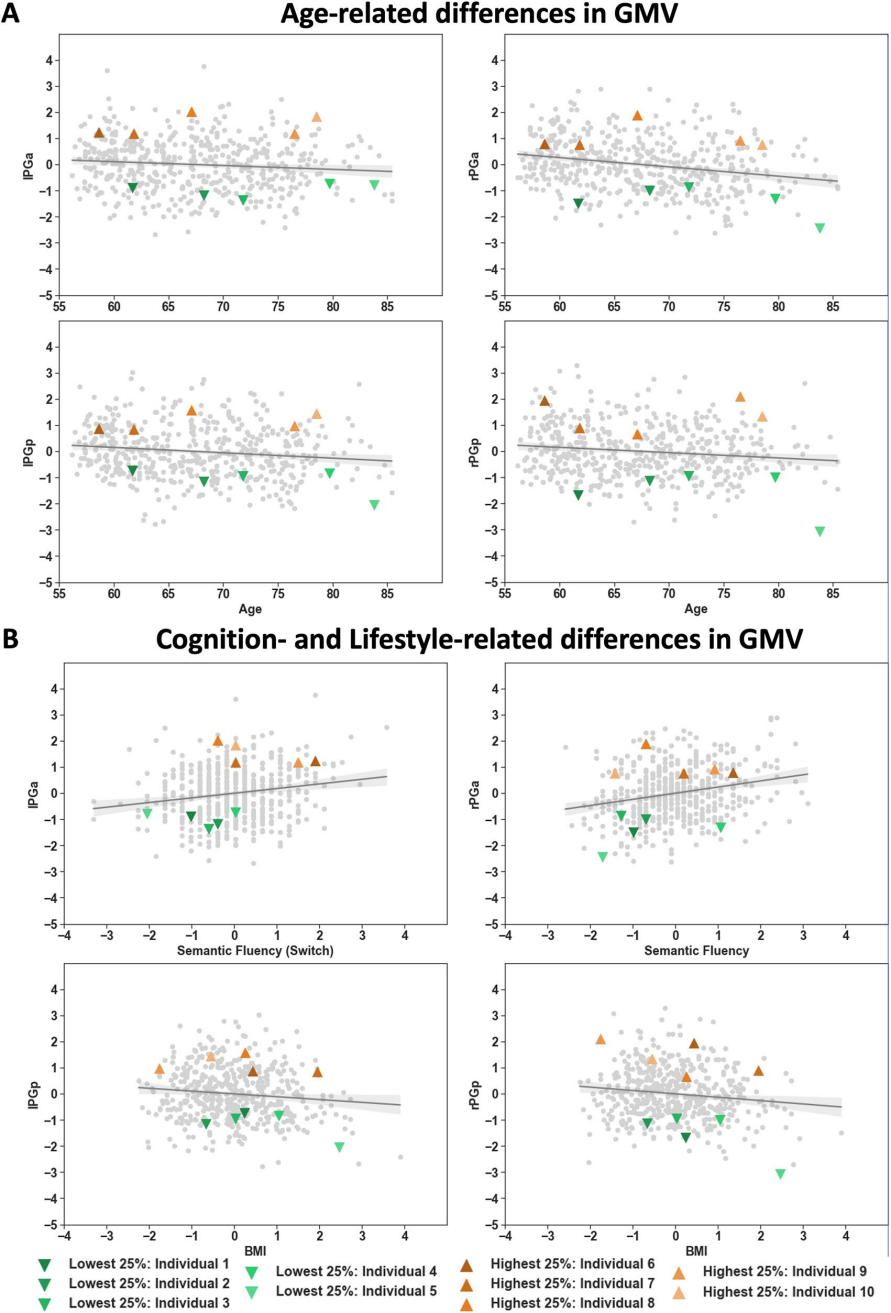
**A** Age-related differences in GMV (*z*-scores) for areas lPGa, rPGa, lPGp and rPGp. **B** Cognitive performance (*z*-scores) and lifestyle-related differences in GMV (*z*-scores). The whole group is represented by gray dots including regression line and confidence interval. Selected individuals within the highest and lowest 25% are marked in orange and green triangles, respectively

Using multiple regression analyses, we examined the associations between GMV of the left and right PGa and PGp and age (while adjusting for sex, education and TBV). We found age-related decreases in GMV for all four parts of the AG, with the highest age-related decrease for right PGa and the lowest decrease for the left PGa (Fig. 7A, Table 2).

**Table 2:** Multiple regression models (inclusion) with GMV of the regions of interest as dependent variables (left and right PGa and PGp) and age, sex, education and TBV as predictors

	lPGa	rPGa	lPGp	rPGp
Age	−0.092 (0.023)	−0.226 (<0.001)	−0.121 (0.001)	−0.131 (<0.001)
Sex	−0.066 (0.167)	−0.036 (0.441)	−0.026 (0.562)	−0.116 (0.007)
Education	0.019 (0.107)	0.034 (0.402)	0.038 (0.335)	0.009 (0.811)
TBV	0.422 (<0.001)	0.447 (<0.001)	0.525 (<0.001)	0.524 (<0.001)

The models include all predictors with standardized regression coefficients and *p*-values in brackets. *TBV* total brain volume

For associations between GMV and behavioral factors, several different forward-selection multiple regression analyses were performed. Regarding cognitive performance, we found relations between GMV of left PGa and figural and semantic verbal fluency, as well as verbal WM. In turn, GMV of left PGp correlated with semantic verbal fluency and visual WM. GMV of right PGa correlated with semantic and phonematic verbal fluency as well as with reasoning, while GMV of right PGp correlated with processing speed. Thus, in both hemispheres, the AG subdivisions correlated with partially distinct cognitive functions in the older adult population. While GMV of all regions of interest but the right PGp were related to semantic verbal fluency, we additionally found the two posterior regions (left and right PGp) to be related to visual WM and (visual) processing speed (for exemplary scatterplots, see Fig. 7B; for all scatterplots, see supplementary Figures S1–4, for regression coefficients, see Table 3).

**Table 3:** Multiple regression models (forward-selection) with GMV of the regions of interest as dependent variables (left and right PGa and PGp) and A) cognitive performance test scores and B) lifestyle variables as predictors

A	lPGa		rPGa		lPGp		rPGp	
Cognition	TBV	0.445 (<.001)	TBV	0.436 (<.001)	TBV	0.533 (<.001)	TBV	0.521 (<.001)
	Figural fluency	0.111 (0.013)	Age	−0.158 (<.001)	Semantic Fluency	0.088 (0.024)	Age	−0.103 (0.009)
	Semantic fluency switch	0.118 (0.01)	Semantic fluency	0.192 (<.001)	Visual WM	0.085 (0.034)	Sex	−0.134 (0.002)
	Verbal WM	−0.093 (0.031)	Phonematic fluency switch	−0.135 (0.005)			Processing speed	−0.08 (0.043)
			Reasoning	0.114 (0.01)				

B	lPGa		rPGa		lPGp		rPGp	
Lifestyle	TBV	0.457 (<.001)	TBV	0.475 (<.001)	TBV	0.550 (<.001)	TBV	0.524 (<.001)
	BMI	−0.131 (0.001)	Age	−0.215 (<.001)	Age	−0.111 (0.003)	Age	−0.136 (<.001)
			Sports	0.093 (0.017)	BMI	−0.078 (0.039)	Sex	−0.143 (0.001)
							BMI	−0.104 (0.004)
							Alcohol	−.099 (0.009)
							Social integration	0.080 (0.025)

All models additionally include covariates of non-interest (age, sex, education, TBV). The models include all significant predictors with standardized regression coefficients and *p*-values in brackets. *TBV* total brain volume; *WM* working memory; *BMI* body mass index

In terms of the association between GMV and lifestyle, multiple regressions revealed left hemispheric parts of the AG (PGa and PGp) to be related to BMI, i.e., higher BMI being related to lower GMV. Additionally, GMV of right area PGa correlated positively with sports. Further, GMV of right area PGp was negatively related to BMI and alcohol consumption and positively to social integration. Thus, there seems to be an overall relation between GMV of the AG and BMI (except for right area PGa), while sports, alcohol consumption and social integration were rather specifically related to GMV of distinct AG sub-regions (for exemplary scatterplots, see Fig. 7B; for all scatterplots, see supplementary Figures S1–4, for regression coefficients, see Table 3).

### Group trends versus individual subjects

**Fig. 8:**
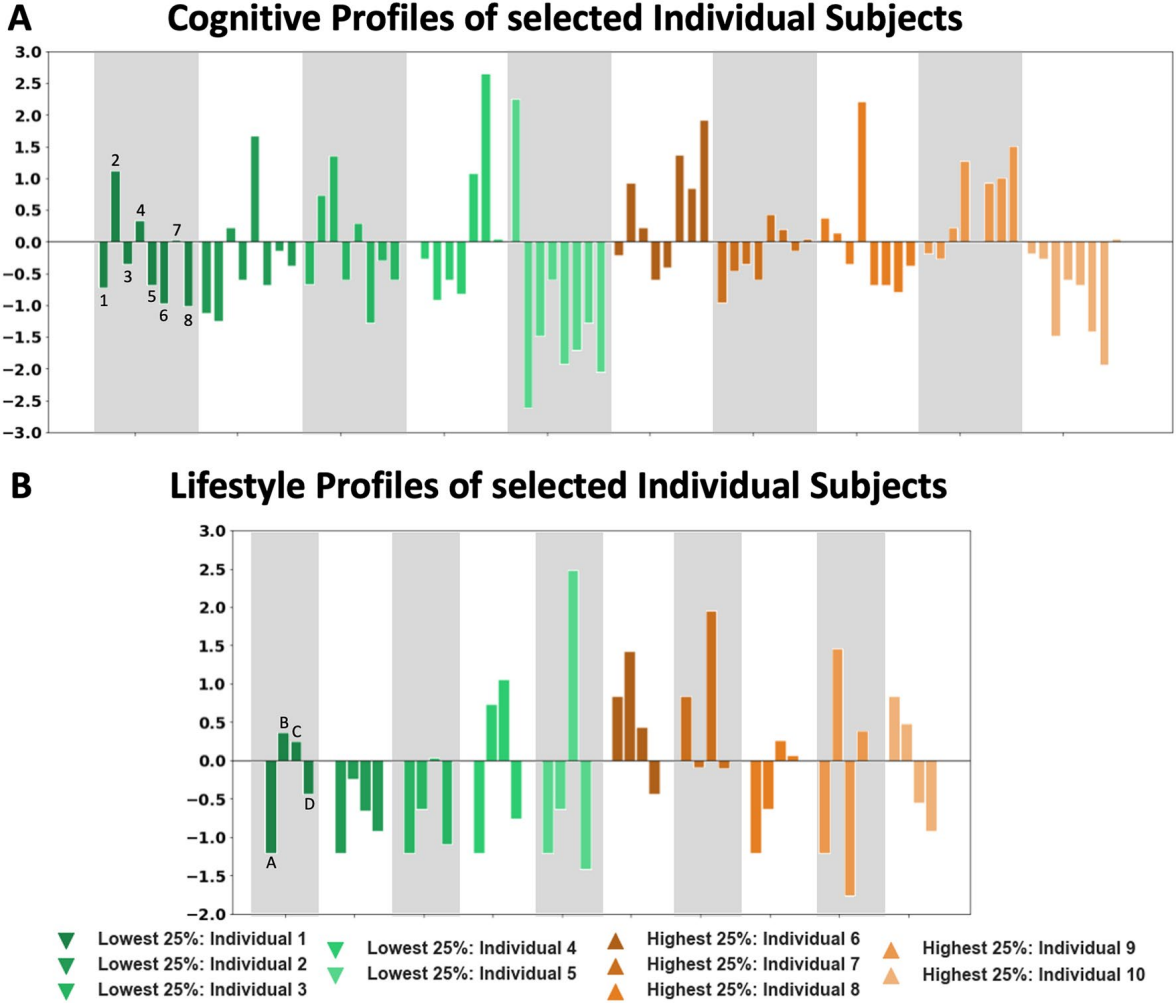
Comparison of selected individuals regarding their **A** cognitive performance (1 = Processing Speed – 2 = Reasoning – 3 = Visual WM – 4 = Verbal WM – 5 = Figural Fluency – 6 = Semantic Fluency – 7 = Phonematic Verbal Fluency Switch – 8 = Semantic Verbal Fluency Switch) and **B** lifestyle habits (A = Alcohol (yes-no) – B = Sports – C = BMI – D = Social Integration Index) for those variables showing significant influences on GMV of any of the AG subdivisions. All cognitive and lifestyle variables were standardized to facilitate comparability across variables

To go beyond group level insights, we addressed the “individual view” bearing in mind the variability between GMV and cognitive abilities and lifestyle habits. We therefore targeted exemplary individual subjects regarding their specific ‘multilevel AG profile’ among those with either highest or lowest GMV (25% percentile groups). Scatterplots shown in Fig. 7A (age-related differences in GMV) and B (exemplary relations between AG GMV and cognitive performance or lifestyle habits, for all other scatterplots, see supplementary Figures S1–4) illustrate the selected subjects in the frame of the here examined sample. While subjects with, e.g., similarly low GMV already cover the whole age range, we additionally looked at their individual cognitive performance and lifestyle profiles (Figure 8A, B). Comparing two individuals with a low GMV in all AG subdivisions, revealed additional differences: In terms of cognitive functioning, individual #3 performs above average in reasoning and visual WM and below average in terms of semantic fluency. At the same time, individual #3 shows a slightly lower social integration index. In contrast, individual #4 shows an above average performance in semantic and phonematic fluency together with a slightly above average BMI. When selecting two subjects with high GMV in the AG, we see a similar heterogeneous picture: Individual #5 shows a high performance in the semantic fluency task, and an above average engagement in sports. In turn, individual #7 shows a high performance in verbal WM together with all lifestyle variables being within the normal range (*z*-scores within 1 SD). Finally, comparing individual #5 with individual #10, both subjects perform low in most of the cognitive tasks presented. However, individual #5 is one of the subjects with a low GMV, while individual #10 exhibited a high GMV. Thus, these individual profiles demonstrate that each individual shows its own cognitive and lifestyle fingerprint, with differential effects for different factors, not reflected by the general group trends.

## Discussion

Aim of the current study was to characterize multimodal brain–phenotype relationships of the AG sub-regions in the older brain. Thereby, we first examined the GMV of the AG sub-regions and the relation to multilevel information about GMV, RSFC and SC of the rest of the brain. We additionally made use of the multilevel atlas framework EBRAINS to enrich the here established results by molecular, genetic and cellular information of these brain areas, to finally obtain a holistic understanding of the cyto-architectonically defined sub-regions, PGa and PGp. In a second step, we conducted group analyses of AG subdivisions in light of age, cognitive performance and lifestyle in older subjects using multimodal sources of information. We finally switched the perspective toward the “individual” to carve out the peculiarities that individual profiles of this multimodal picture of the AG might reveal in contrast to the insight based on group-level inference, an essential aspect when it comes to medical conditions and treatment considerations.

### Multimodal characterization AG sub-regions

With respect to brain–brain relationships, in the current study, we performed regression analyses to examine the associations between GMV of the AG subdivisions and a) GMV of, and b) RSFC, and c) SC with all parcels of the Julich-Brain Atlas. The resulting patterns of these brain–brain relationships were found to be different for PGa and PGp as well as across modalities. With this, the current results support the notion of previous studies, reporting the AG to consist of different subdivisions (Caspers et al. 2006, 2008) and emphasize the need to explore these individually. We here extended these observations by showing that the AG subdivisions exhibit individual spatial patterns of covariance for all three modalities investigated (GMV, RSFC and SC). Thus, with the current results based on an older adult population, we show multimodal evidence for a clear distinction of areas PGa and PGp in the AG.

Importantly, it has to be mentioned that spatial association patterns were not only heterogeneous across the regions of interest, but also across the three modalities (GMV, RSFC, SC) within one ROI. In accordance with the notion of brain plasticity in even healthy older adults (for a review, see Reuter-Lorenz and Park 2014), differences in GMV of the AG subdivisions seem to, at least in part, affect GMV, RSFC and SC in a distinct way. For instance, we found GMV of left areas PGa and PGp to be associated with GMV of the left hemispheric parietal and dorsolateral prefrontal brain areas, possibly reflecting a frontoparietal network, involved in executive functions and working memory (Yeo et al. 2011; Smith et al. 2009). Interestingly, focusing on left area PGp, we also found a frontoparietal association in terms of RSFC. Here, a lower GMV was related to higher RSFC between this area and the dorsolateral prefrontal cortex of the contralateral hemisphere. Aging studies, so far, have shown that a decrease in GMV in posterior brain regions might be related to a higher functional connections to frontal brain regions, the so-called posterior to anterior shift in aging (Dolcos et al. 2002). These effects shown here are in line with this and might represent a compensatory mechanism of the brain, to maintain cognitive performance as stable as possible in our older adult population. Furthermore, while the frontoparietal network is supposed to be a mainly lateralized brain network, we here found a GMV dependent difference in RSFC with the dorsolateral prefrontal cortex of the contralateral hemisphere. With regard to the aging population and plasticity of the older adult brain, an increase in communication between the two hemispheres might reflect similar compensatory attempts for structural brain atrophy as discussed above to maintain cognitive performance as stable as possible [HAROLD, Cabeza et al. (2002); Jockwitz et al. (2017); Reuter-Lorenz and Lustig (2005); Holler-Wallscheid et al. (2017))]. Thus, the current results might, at least in part, represent multimodal evidence for the functionally derived aging theories in the older population.

In addition, both (left and right) areas PGp showed an association to GMV of the visual cortex. This fits to the results in terms of GMV patterns as well as in terms of SC, i.e., connectivity between areas PGp and the visual cortex in both analyses. Furthermore, Caspers et al. (2013) reported similar receptor distributions between area PGp and the visual cortex, which is in line with the current observations in terms of GMV and SC. The interaction of AG and the visual cortex, also known as dorsal visual stream, is essential for an intact visuomotor system and has already been reported to be age-sensitive (Yamasaki et al. 2012; Sciberras-Lim and Lambert 2017; Wu et al. 2020); i.e., substantially higher gray matter reduction for the dorsal visual stream as compared to, e.g., the ventral visual stream (Ziegler et al. 2012). This multimodal perspective supports previous notions that area PGp might be closely linked to higher-order visual processing, also during older ages.

### Group analyses of AG subdivisions in light of age, cognitive performance and lifestyle

Based on the multimodal group results, the AG subdivisions can be characterized by different properties in terms of gray matter, functional and structural connectivity. In terms of brain–phenotype relationships on the group level, we additionally found differential associations for the AG subdivisions. We found the most pronounced age-related GMV decreases in right PGa, followed by right PGp, left PGp and finally left PGa. Asymmetric differences concerning age-related decreases in brain structure have been previously reported, also for the inferior parietal lobule and the AG itself (e.g., (Plessen et al. 2014; Jockwitz et al. 2017; Roe et al. 2021). This is in line with the so-called right hemi aging theory (Grady et al. 1994) stating that the right hemisphere, mainly responsible for visuospatial functions, declines earlier as compared to the left hemisphere. In a previous analysis, we could already establish that this rather global statement also holds true for the right versus left AG, at least in terms of cortical folding indices (Jockwitz et al. 2017). We now could verify this effect also regarding GMV.

In terms of cognitive performance, previous studies reported the right AG to be associated with visual spatial attention, calculations, or self-processing (Corbetta and Shulman 2002; Arsalidou and Taylor 2011; Seghier 2013), while the left AG is rather involved in language functions, especially semantic processing and memory (Seghier 2013; Heim et al. 2019). In the current study, however, we revealed associations between semantic verbal fluency and GMV of both, left PGa/PGp and right PGa. This is only partially in line with and rather extends results obtained by a large meta-analysis investigating the semantic system in the brain (Binder et al. 2009). While Binder et al. (2009) showed a lateralization of semantic language processing to the left hemisphere, we here showed a bilateral relation between GMV and verbal fluency in the older adult population. In terms of semantic language processing, this performance-dependent GMV might serve as a structural correlate for the so-called HAROLD model (Cabeza et al. 2002), stating that older in comparison to younger adults recruit bilateral brain networks to maintain cognitive functions as stable as possible.

Interestingly, additionally focusing on the genetic information extracted from EBRAINS, we found differential language-related gene expressions in areas PGa versus PGp. Generally, area PGa shows a higher expression of ATP2C2 (with higher right as compared to left hemispheric expression) and a lower expression of FOXP2 as compared to area PGp. Since both genes are supposed to support successful language processing during the lifespan [i.e., FOXP2 is supposed to be involved in the development of speech and language, ATP2C2 has been associated with dyslexia and other communication disorders (Lai et al. 2003; Newbury and Monaco 2010; Lambert et al. 2011; Unger et al. 2021a, b)], the difference in gene expression may suggest a functional diversity to exist between areas PGa and PGp. These results, indeed, align with the functional diversity found between these areas, especially in the right hemispheric AG, i.e., area PGa is related to semantic fluency, whereas area PGp shows no correlation with semantic fluency. Emphasizing that the current study focused on the older adult brain, it needs to be highlighted that the right hemisphere is supposed to be more age-sensitive as compared to the left hemisphere. Thus, we here might unravel gene-dependent differences in the two areas, which might become particularly functionally relevant during older ages, when aging effects on brain structure already start to unveil.

Differences with respect to the brain–behavior relationships, however, were not only present within the right AG. Rather, we found verbal WM to be related to GMV of left PGa and visual WM to be related to GMV in left PGp. Generally, the current results are in accordance with functional connectivity-based results showing an involvement of the (left) AG during WM performance (Smith et al. 2009; Rottschy et al. 2012; Vatansever et al. 2016; Marek and Dosenbach 2018; Yao et al. 2020). As already shown for verbal fluency, at least in the older adult population, there might be a regional difference in the involvement of left AG in WM, with left area PGa being related to verbal WM and left area PGp being related to visual WM. Here, it is particularly useful to additionally incorporate information from the EBRAINS multilevel platform that supports the results found in the current sample of older adults. For instance, receptor fingerprints of the AG subdivisions show that especially the more posterior lying brain regions PGp bilaterally have receptor fingerprints similar to extra-striate cortices, which is in line with the current results.

These insights were supplemented by differences in lifestyle habits in association with age-related AG subregion volume differences. We found a higher BMI to be associated with lower GMV in all areas except right area PGa. A negative association between widespread brain structure and BMI has been established in several studies (e.g., Taki et al. 2008; Kharabian Masouleh et al. 2020). Some studies showed a particular association with the inferior parietal lobule. For instance, Kurth et al. (2013) showed a negative association between BMI and GMV of the inferior parietal lobule and Cheke et al. (2016) reported reduced functional activity during an episodic memory task in the AG, which could be explained by reductions in brains structure, as found in the current study.

Furthermore, we found GMV of the two right hemispheric AG parts to be associated with several other lifestyle variables. GMV of right area PGa was positively associated to sports and GMV of the right area PGp was negatively correlated with alcohol consumption and positively correlated to social integration. Sports and social integration have been shown to be beneficial in terms of gray matter structure during the aging process (Erickson et al. 2015; Bittner et al. 2019, 2021; Domingos et al. 2021). Particularly interesting, we found a relation between structural atrophy of area PGp and the social integration index. With the right AG being associated with social cognition (Bitsch et al. 2018) and social integration (Park et al. 2021), the current results might hint at a special role of right area PGp in social behavior during older ages.

Taken together, results derived from a large population-based sample of older adults reveal quite heterogenous patterns for left and right areas PGa and PGp. We found a mixed picture of age differences in terms of GMV, together with differential relations to cognitive performance and lifestyle. Together with the integration of information derived from EBRAINS, we highlight the need to investigate the AG subdivisions as different entities, rather than one macro-anatomical structure, which might obscure specific relations between brain regional architecture and behavior and cognition.

### Individual profiles

An important aim of the current study was to not only characterize global alterations of areas PGa and PGp in the older adult population, but to also investigate individual profiles of subjects showing particularly high or low GMV of the AG subdivisions as an example of relevant deviations from global trends typically reported in group-level analyses. The question of the transition from the group level to the individual subject tackles one of the most important topics when it comes to modern neuroscience including precision medicine. By amending the group results with individual subject profiles in the present study, we aimed at gaining further awareness of this topic in the neuroscientific community.

For instance, the group results suggested that lower GMV would be related to lower performance in the verbal fluency tasks, together with a higher BMI. Looking at one of the individuals who showed low GMV in all AG subdivisions, we see that this individual #4 shows an above average performance in semantic and phonematic fluency together with a slightly above average BMI. Similarly, comparing two subjects, one showing low GMV of the AG (individual #5) and the other one showing high GMV (individual #10), both subjects perform low in most of the cognitive tasks presented. Thus, bearing in mind the variability between GMV and cognitive abilities and lifestyle habits (cf. Fig. 7A and B) when examining this individual, e.g., in case of a medical examination, group trends could not easily be applied to this specific individual as some might fit more or less, while for other factors, there is considerable and differential deviation. Rather, these individual profiles demonstrate that each individual shows its own cognitive and lifestyle fingerprint, that is not necessarily reflected by the group effects. In fact, results from the current data suggest that individuals and their cognitive/lifestyle profiles may largely deviate from estimated group trends leading to the question what this would mean for future research in the field of cognitive neuroscience.

Group analyses aim at extracting general principles of brain–behavior relationships, e.g., the “average” association between GMV decrease and cognitive performance decline in older adults. Applying this “one size fits all” approach to clinical cases, e.g., a group of Alzheimer patients, and relying on this principle would mean that all patients would be treated the same way (Reitz 2016). However, from previous work, it is well known that neurodegenerative diseases might show individual peculiarities, where not every patient exhibits the same symptoms. Likewise, not every patient responds to the same treatment (Reitz 2016). The here presented results between the poles of group results and individual cognitive/lifestyle profiles tap into this conflict, by showing that even in a normal older adult population, each subject has his/her own individual fingerprint of brain and behavioral particularities. Although principles derived from group-level analyses, of course, build a guideline for the average subject or patient, the current individual dissimilarities stress that a characterization at the individual level, in contrast to group averages, will be an inevitable step toward successful diagnostics and treatments (Reitz 2016; Zimmermann et al. 2016). Importantly, individual characterizations require rich datasets, including information on brain and behavior at different levels, such as molecular and genetics. Since this kind of information might not be accessible to every research group, the EBRAINS (Amunts et al. 2016) interactive tool combining multilevel data from various sources enables deep multifaceted characterizations of the brain at multiple level in one common framework. In the current study, we used EBRAINS to support the brain–behavior relationships presented here with both genetic and molecular findings to obtain a holistic characterization of the AG subdivisions.

## Conclusion

Based on the multimodal group results, the AG can be considered as a structure heterogeneously affected in the aged brain: First, GMV, RSFC, and SC patterns provide multimodal evidence that the AG subdivisions seem to be involved in different brain networks sub-serving distinct cognitive functions, which could further be supported by integrating molecular and genetic information from EBRAINS. Second, age differentially affected GMV of the AG sub-divisions, with the highest GMV decrease in rPGa. Third, the different AG parts showed distinct associations with cognitive abilities or lifestyle habits hinting at a functional specificity of each region. However, the individual profiles show that the relations identified at the group level are not necessarily transferable to the individual level. Hence, general observations within the older adult population need to be carefully considered, especially when it comes to the assessment and treatment of individual patients.

## Supplementary Information

Below is the link to the electronic supplementary material.

Supplementary file1 (PPTX 1311 KB)

Supplementary file2 (DOCX 31 KB)

## Data Availability

Due to local regulations of data acquisition and use, data of 1000BRAINS are available upon request from the responsible PI.
