# Supplementary material for: Characterization of the angular gyrus in an older adult population: a multimodal multilevel approach
Source: Brain Struct Funct. 2022 Jul 29;228(1):83–102. doi: 10.1007/s00429-022-02529-3 (PMC9813183; doi:10.1007/s00429-022-02529-3)
Supplement: Supplementary file 1 — Supplementary file1 (PPTX 1311 KB) [file 429_2022_2529_MOESM1_ESM.pptx]

## Slide 1
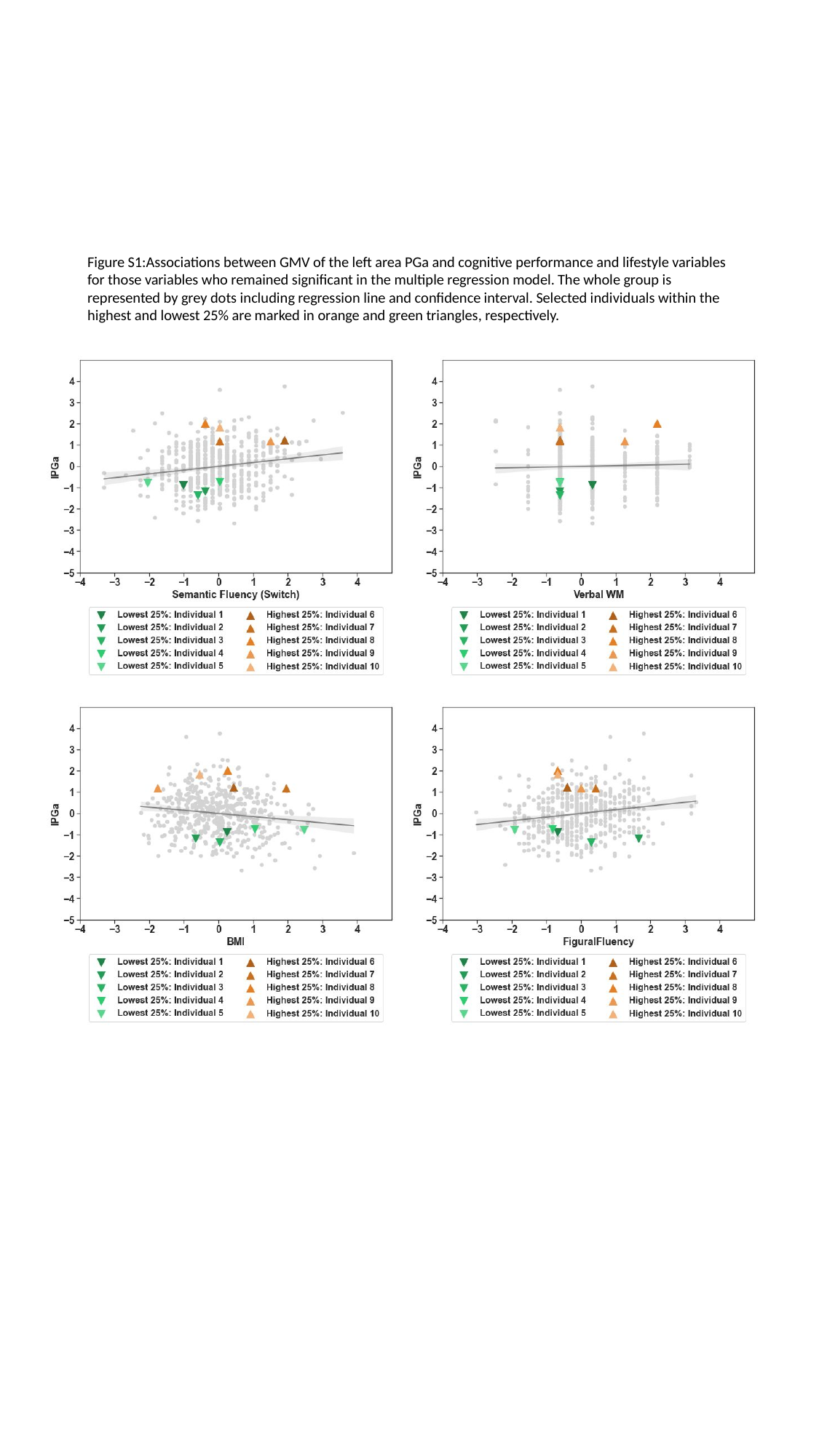

Figure S1:Associations between GMV of the left area PGa and cognitive performance and lifestyle variables for those variables who remained significant in the multiple regression model. The whole group is represented by grey dots including regression line and confidence interval. Selected individuals within the highest and lowest 25% are marked in orange and green triangles, respectively.

## Slide 2
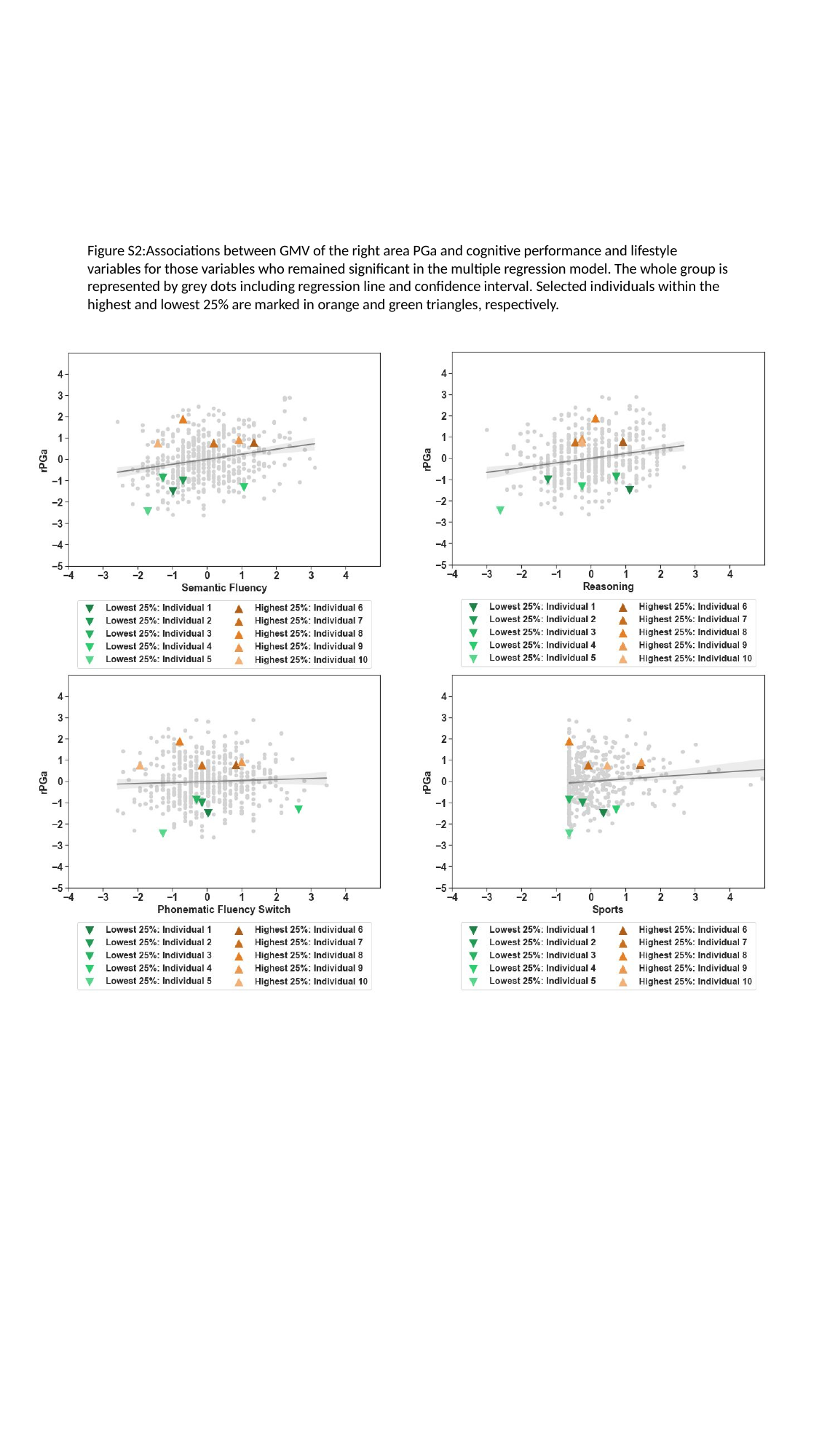

Figure S2:Associations between GMV of the right area PGa and cognitive performance and lifestyle variables for those variables who remained significant in the multiple regression model. The whole group is represented by grey dots including regression line and confidence interval. Selected individuals within the highest and lowest 25% are marked in orange and green triangles, respectively.

## Slide 3
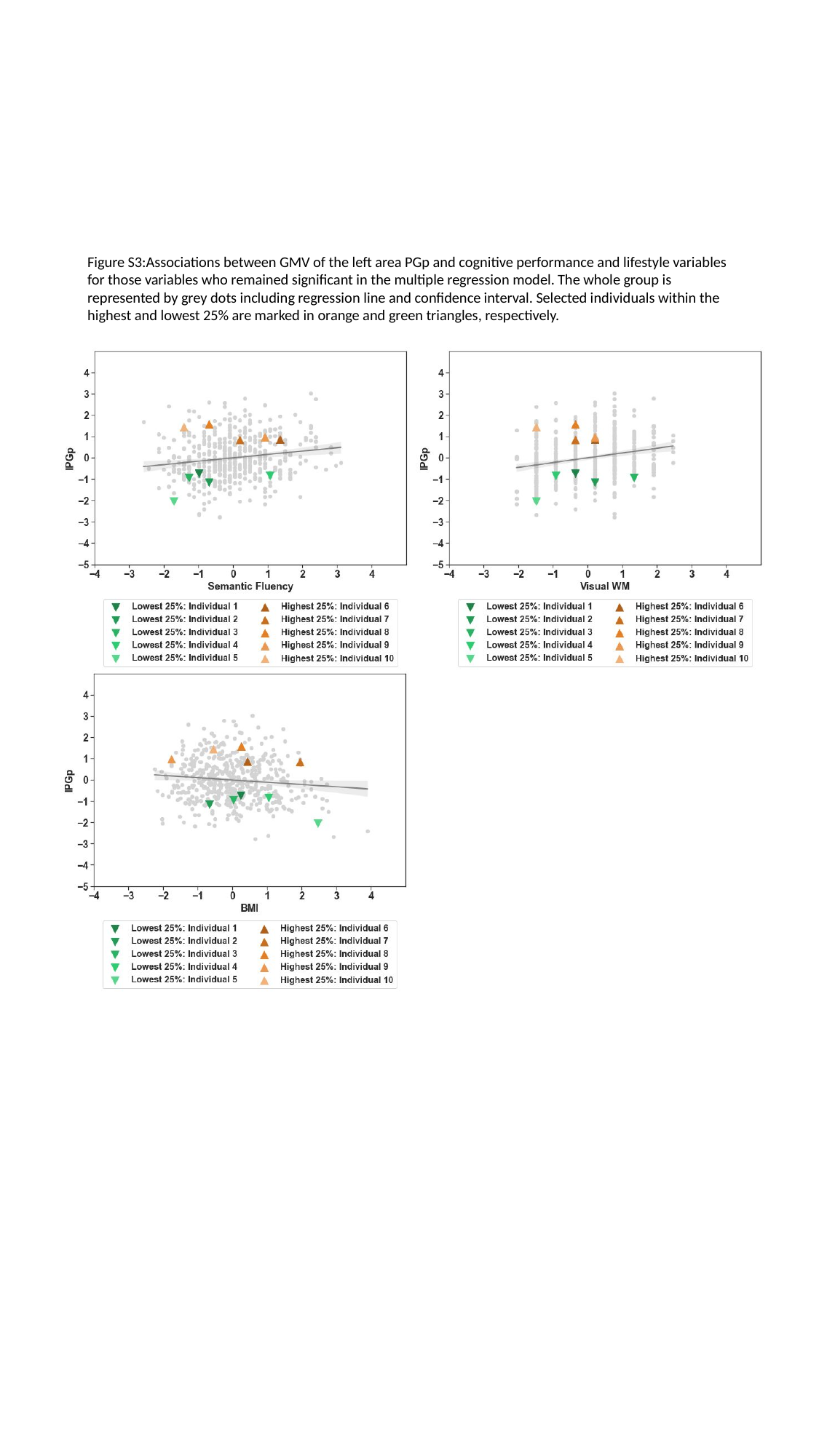

Figure S3:Associations between GMV of the left area PGp and cognitive performance and lifestyle variables for those variables who remained significant in the multiple regression model. The whole group is represented by grey dots including regression line and confidence interval. Selected individuals within the highest and lowest 25% are marked in orange and green triangles, respectively.

## Slide 4
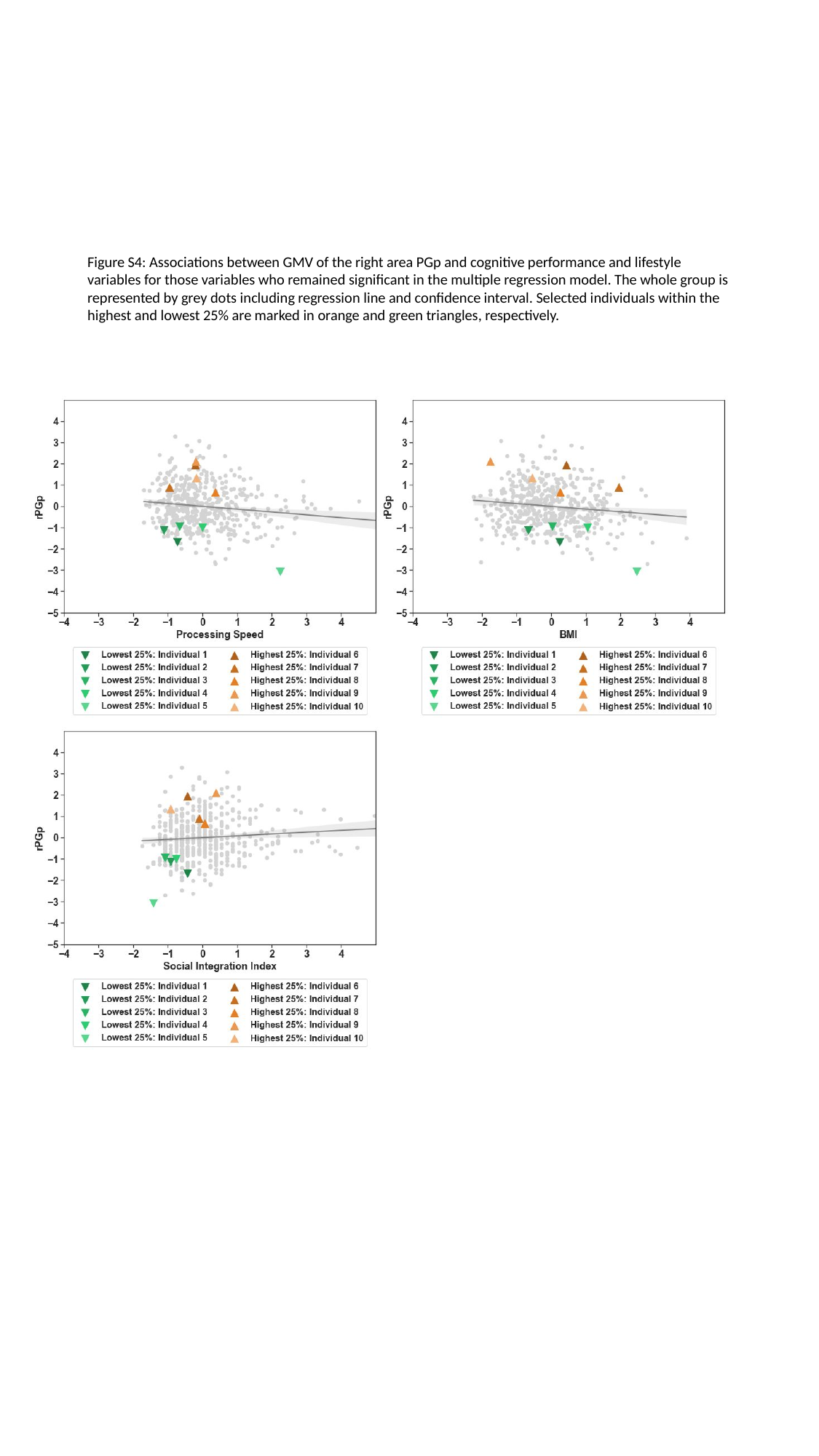

Figure S4: Associations between GMV of the right area PGp and cognitive performance and lifestyle variables for those variables who remained significant in the multiple regression model. The whole group is represented by grey dots including regression line and confidence interval. Selected individuals within the highest and lowest 25% are marked in orange and green triangles, respectively.

## Slide 5
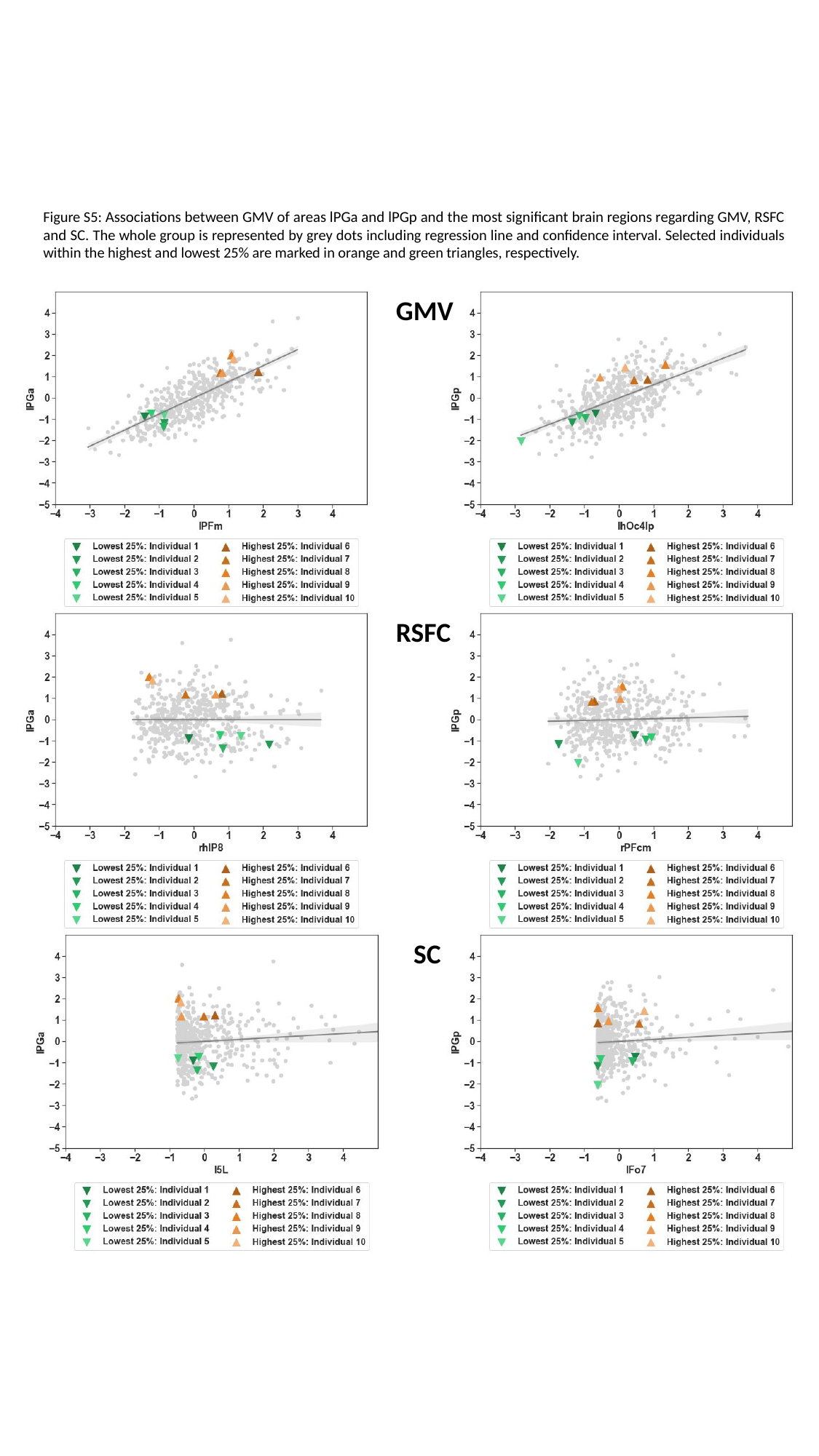

Figure S5: Associations between GMV of areas lPGa and lPGp and the most significant brain regions regarding GMV, RSFC and SC. The whole group is represented by grey dots including regression line and confidence interval. Selected individuals within the highest and lowest 25% are marked in orange and green triangles, respectively.
GMV
RSFC
SC
