# Supplementary material for: Characterization of the angular gyrus in an older adult population: a multimodal multilevel approach
Source: Brain Struct Funct. 2022 Jul 29;228(1):83–102. doi: 10.1007/s00429-022-02529-3 (PMC9813183; doi:10.1007/s00429-022-02529-3)
Supplement: Supplementary file 2 — Supplementary file2 (DOCX 31 KB) [file 429_2022_2529_MOESM2_ESM.docx]

Table S1: Multiple regression models (forward-selection) with GMV of the regions of interest as dependent variables (left and right PGa and PGp) and GMV of all areas belonging to the Julich-Brain atlas as predictors. All models additionally include covariates of non-interest (age, sex, education, TBV). The models include all significant predictors with standardized regression coefficients and p-values in brackets. BF = basal forebrain, FOperc = frontal operculum, FusG = Fusiform gyrus, HC = Hippocampus, IPL = inferior parietal lobule, IPS = intraparietal sulcus, LOC = lateral occipital cortex, OFC = Orbitofrontal cortex, POperc, Parietal operculum, PreCG = Precentral gyrus, SPL = superior parietal lobule, SMA = supplementary motor area, STG = Superior temporal gyrus, IFG = inferior frontal gyrus, pACC = pregenual anterior cingulate cortex, POS =parieto-occipital sulcus, PostCG = Postcentral gyrus, V1 = primary visual cortex, V2 = secondary visual cortex, TBV = Total brain volume.

| left PGa | | right PGa | | left PGp | | right PGp | |
| --- | --- | --- | --- | --- | --- | --- | --- |
| lCh123 (BF) | -0.078 (0.02) | *rMF (Amygdala)* | 0.07 (0.019) | *rCM (Amygdala)* | -0.075 (0.01) | *lhOc1 (V1)* | -0.23 (<.001) |
| rFo3 (FOperc) | 0.155 (<.001) | *rFG4 (FusG)* | 0.108 (0.007) | *rMF (Amygdala)* | 0.078 (0.004) | *rFrontaltoTemporal (GapMap)* | -0.227 (<.001) |
| lFG4 (FusG) | -0.071 (0.064) | *lFrontaltoOccipital (GapMap)* | 0.127 (0.026) | *lhOc4d (Cuneus)* | -0.151 (<.001) | *rPFm (IPL)* | -0.184 (<.001) |
| lFrontalII (GapMap) | -0.096 (0.012) | *lTemporaltoParietal (GapMap)* | 0.144 (0.018) | *lFo5 (FOperc)* | -0.136 (<.001) | *rTI (STG)* | -0.124 (0.011) |
| rTemporaltoParietal (GapMap) | 0.185 (0.001) | *rIg1 (Insula)* | -0.14 (0.001) | *lFo6 (FOperc)* | 0.084 (0.025) | *lTE1.2 (STG)* | -0.103 (0.002) |
| rHCProsubiculum (HC) | 0.105 (0.001) | *lIF (Insula)* | -0.067 (0.02) | *rFo5 (FOperc)* | 0.084 (0.011) | *l5M (SPL)* | -0.072 (0.019) |
| lPFcm (IPL) | -0.16 (<.001) | *rPFcm (IPL)* | -0.097 (0.024) | *lFG4 (FusG)* | 0.145 (<.001) | *lhIP4 (IPS)* | -0.069 (0.018) |
| lPGp (IPL) | 0.213 (<.001) | *rPGp (IPL)* | 0.214 (<.001) | *lFrontalII (GapMap)* | -0.102 (0.008) | *lhOc5 (LOC)* | 0.064 (0.013) |
| lPFm (IPL) | 0.6 (<.001) | *rPFm (IPL)* | 0.367 (<.001) | *lFrontaltoOccipital (GapMap)* | 0.163 (0.003) | *lIa (Insula)* | 0.064 (0.031) |
| lhIP6 (IPS) | -0.096 (<.001) | *rhIP4 (IPS)* | -0.215 (<.001) | *rTemporaltoParietal (GapMap)* | 0.187 (0.002) | *rId6 (Insula)* | 0.07 (0.038) |
| rhIP7 (IPS) | 0.111 (<.001) | *rhIP6 (IPS)* | 0.115 (<.001) | *rCA1 (HC)* | -0.104 (0.009) | *lCh4 (BF)* | 0.074 (0.014) |
| rhIP2 (IPS) | 0.131 (<.001) | *rhOc4la (LOC)* | -0.225 (<.001) | *lHCPresubiculum (HC)* | 0.074 (0.015) | *rFo4 (Foperc)* | 0.079 (0.016) |
| lhOc4v (LOC) | -0.088 (0.026) | *lhOc4lp (LOC)* | 0.142 (<.001) | *lCA3 (HC)* | 0.104 (0.002) | *lPGa (IPL)* | 0.087 (0.008) |
| rFo2 (OFC) | -0.095 (0.007) | *lOP4 (Poperc)* | 0.07 (0.046) | *lFG3 (IFG)* | -0.106 (0.008) | *l6mp (SMA)* | 0.087 (0.007) |
| rFo7 (OFC) | -0.076 (0.044) | *l6mp (SMA)* | -0.108 (0.002) | *rIF (Insula)* | -0.053 (0.034) | *lOP4 (Poperc)* | 0.117 (<.001) |
| rOP3 (POperc) | 0.103 (0.003) | *l7P (SPL)* | 0.173 (<.001) | *rPFm (IPL)* | 0.103 (0.001) | *rhOc4la (LOC)* | 0.12 (0.006) |
| l4a (PreCG) | 0.104 (0.001) | *rTE2.2 (STG)* | 0.164 (<.001) | *rPGp (IPL)* | 0.198 (<.001) | *lhOc2 (V2)* | 0.126 (0.033) |
| l5Ci (SPL) | -0.106 (<.001) | *Age* | -0.062 (0.04) | *lPGa (IPL)* | 0.216 (<.001) | *rPGa (IPL)* | 0.14 (<.001) |
|  |  |  |  | *lhIP4 (IPS)* | 0.16 (<.001) | *rTeI (STG)* | 0.196 (<.001) |
|  |  |  |  | *lhOc4lp (LOC)* | 0.235 (<.001) | *rTemporaltoParietal (GapMap)* | 0.197 (0.001) |
|  |  |  |  | *lp24c (pACC)* | -0.065 (0.04) | *rhIP4 (IPS)* | 0.209 (<.001) |
|  |  |  |  | *lp32 (pACC)* | 0.145 (<.001) | *rhOc4lp (LOC)* | 0.214 (<.001) |
|  |  |  |  | *lOP3 (Poperc)* | -0.157 (<.001) | *lPGp (IPL)* | 0.256 (<.001) |
|  |  |  |  | *lOP6 (Poperc)* | 0.092 (0.006) |  |  |
|  |  |  |  | *rhOc6 (POS)* | 0.084 (0.006) |  |  |
|  |  |  |  | *l1 (PostCG)* | -0.088 (0.019) |  |  |
|  |  |  |  | *l2 (PostCG)* | -0.069 (0.057) |  |  |
|  |  |  |  | *r1 (PostCG)* | 0.122 (<.001) |  |  |
|  |  |  |  | *l7P (SPL)* | -0.113 (<.001) |  |  |
|  |  |  |  | *r5L (SPL)* | 0.08 (0.006) |  |  |
|  |  |  |  | *lTE2.2 (STG)* | -0.08 (0.019) |  |  |
|  |  |  |  | *rhOc1 (V1)* | -0.085 (0.014) |  |  |

Table S2: Multiple regression models (forward-selection) with GMV of the regions of interest as dependent variables (left and right PGa and PGp) and RSFC of all areas belonging to the Julich-Brain atlas as predictors. All models additionally include covariates of non-interest (age, sex, education, TBV). The models include all significant predictors with standardized regression coefficients and p-values in brackets. IPS = intraparietal sulcus, SPL = superior parietal lobule, Foperc = Frontal operculum, FusG = Fusiform gyrus, Poperc = Parietal Operculum, IPL = inferior parietal lobule, STG = Superior temporal gyrus, HC = Hippocampus, OFC = Orbitofrontal cortex, LOC = Lateral Occipital Cortex, SFS = Superior frontal sulcus, EC = Entorhinal cortex, TBV = Total brain volume.

| left PGa | | right PGa | | | left PGp | | right PGp | |
| --- | --- | --- | --- | --- | --- | --- | --- | --- |
| lhIP4 (IPS) | -0.207 (<.001) | *Age* | -0.205 (<.001) | | *Age* | -0.138 (<.001) | *rId5 (Insula)* | -0.132 (<.001) |
| lId4 (Insula) | -0.14 (<.001) | *rhIP7 (IPS)* | -0.18 (<.001) | | *r7PC (SPL)* | -0.117 (0.003) | *rhOc5 (LOC)* | -0.125 (0.001) |
| r7P (SPL) | -0.133 (0.012) | *lId1 (Insula)* | -0.163 (<.001) | | *lId2 (Insula)* | -0.113 (0.002) | *Age* | -0.123 (<.001) |
| rFo4 (Foperc) | -0.119 (0.003) | *rOP5 (Poperc)* | -0.136 (0.002) | | *lPFm (IPL)* | -0.111 (0.005) | *lPGa (IPL)* | -0.116 (0.01) |
| rFo7 (Foperc) | -0.098 (0.019) | *lFrontaltoOccipital (GapMap)* | -0.081 (0.046) | | *r7M (SPL)* | -0.104 (0.007) | *r7P (SPL)* | -0.115 (0.009) |
| Age | -0.093 (0.017) | *lPFcm (IPL)* | | 0.137 (0.001) | *lOP8 (Poperc)* | -0.097 (0.025) | *Sex* | -0.114 (0.004) |
| l7P (SPL) | -0.089 (0.06) | *rTE3 (STG)* | 0.159 (<.001) | | *rFrontalII (GapMap)* | -0.094 (0.012) | *r6d3 (SFS)* | -0.102 (0.006) |
| rFG1 (FusG) | 0.091 (0.025) | *lCA3 (HC)* | 0.163 (<.001) | | *lFo7 (OFC)* | 0.112 (0.006) | *r5L (SPL)* | -0.086 (0.032) |
| lIa (Insula) | 0.114 (0.005) | *TBV* | 0.476 (<.001) | | *lhIP5 (IPS)* | 0.117 (0.002) | *lhIP6 (IPS)* | -0.082 (0.037) |
| lhIP6 (IPS) | 0.143 (<.001) |  |  | | *l6ma (SMA)* | 0.121 (0.002) | *r7M (SPL)* | -0.065 (0.077) |
| rhIP8 (IPS) | 0.164 (0.003) |  |  | | *rPFcm (IPL)* | 0.146 (<.001) | *rEnt (EC)* | 0.067 (0.062) |
| TBV | 0.435 (<.001) |  |  | | *TBV* | 0.532 (<.001) | *lFo2 (OFC)* | 0.088 (0.011) |
|  |  |  |  | |  |  | *rTemporaltoParietal (GapMap)* | 0.095 (0.009) |
|  |  |  |  | |  |  | *lCA3 (HC)* | 0.097 (0.006) |
|  |  |  |  | |  |  | *r7A (SPL)* | 0.18 (<.001) |
|  |  |  |  | |  |  | *rhIP5 (IPS)* | 0.201 (<.001) |
|  |  |  |  | |  |  | *TBV* | 0.537 (<.001) |

Table S3: Multiple regression models (forward-selection) with GMV of the regions of interest as dependent variables (left and right PGa and PGp) and SC of all areas belonging to the Julich-Brain atlas as predictors. All models additionally include covariates of non-interest (age, sex, education, TBV). The models include all significant predictors with standardized regression coefficients and p-values in brackets. SPL = Superior parietal lobule, LOC = lateral occipital cortex, PostCG = Postcentral gyrus, HC = Hippocampus, FusG = Fusiform gyrus, IPS = Intraparietal sulcus, sACC = subgenual anterior cingulate cortex, IPL = inferior parietal lobule, OFC = Orbitofrontal cortex, Poperc = Parietal Operculum, STG = superior temporal gyrus, POS = parieto-occipital sulcus, SFS = superior frontal sulcus, Foperc = Frontal operculum, FP = Frontal Pole, PreCG = Precentral gyrus, V1 = primary visual cortex, SMA = supplementary motor area, TBV = Total brain volume.

| left PGa | | right PGa | | left PGp | | right PGp | |
| --- | --- | --- | --- | --- | --- | --- | --- |
| rHATA (HC) | -0.082 (0.025) | *rTE2.1 (STG)* | -0.082 (0.025) | *r6d3 (SFS)* | -0.079 (0.047) | *rhIP3 (IPS)* | -0.086 (0.013) |
| Age | -0.089 (0.017) | *rOP3 (POperc)* | -0.085 (0.018) | *lLB (Amygdala)* | -0.088 (0.012) | *Sex* | -0.086 (0.032) |
| l2 (PostCG) | -0.092 (0.014) | *rs24 (sACC)* | -0.089 (0.014) | *rhOc1 (V1)* | -0.089 (0.017) | *lhOc1 (V1)* | -0.09 (0.014) |
| lhOc4lp (LOC) | -0.121 (0.001) | *rhIP5 (IPS)* | -0.119 (0.001) | *lFp2 (FP)* | -0.093 (0.017) | *rhOc4lp (LOC)* | -0.141 (<.001) |
| l7A (SPL) | -0.192 (<.001) | *rhOc4lp (LOC)* | -0.14 (<.001) | *l4a (PreCG)* | -0.093 (0.018) | *rhIP8 (IPS)* | -0.15 (<.001) |
| lFG4 (FusG) | 0.086 (0.024) | *rOP4 (Poperc)* | -0.156 (<.001) | *lFo6 (Foperc)* | -0.094 (0.019) | *Age* | -0.153 (<.001) |
| rhIP7 (IPS) | 0.098 (0.009) | *Age* | -0.2 (<.001) | *l6d3 (SFS)* | -0.102 (0.006) | *rOP4 (Poperc)* | -0.218 (<.001) |
| rs32 (sACC) | 0.122 (<.001) | *rMF (Amygdala)* | 0.073 (0.04) | *Age* | -0.113 (<.001) | *lhIP2 (IPS)* | 0.071 (0.037) |
| lPGp (IPL) | 0.132 (<.001) | *lMF (Amygdala)* | 0.081 (0.023) | *lhPO1 (POS)* | -0.127 (<.001) | *rPGa (IPL)* | 0.072 (0.036) |
| lFo7 (OFC) | 0.139 (<.001) | *rPGp (IPL)* | 0.093 (0.015) | *l7A (SPL)* | -0.163 (<.001) | *rIg2 (Insula)* | 0.088 (0.015) |
| l5L (SPL) | 0.211 (<.001) | *rCA3 (HC)* | 0.109 (0.003) | *l6ma (SMA)* | 0.079 (0.035) | *rhIP5 (IPS)* | 0.089 (0.014) |
| TBV | 0.478 (<.001) | *r5M (SPL)* | 0.122 (0.001) | *rHCProsubiculum (HC)* | 0.097 (0.005) | *r44 (Broca)* | 0.097 (0.015) |
|  |  | *lPGa (IPL)* | 0.127 (<.001) | *l5M (SPL)* | 0.097 (0.006) | *rhOc4la (LOC)* | 0.111 (0.003) |
|  |  | *rhIP1 (IPS)* | 0.143 (<.001) | *lOP7 (Poperc)* | 0.116 (0.002) | *rhIP7 (IPS)* | 0.131 (<.001) |
|  |  | *rOP6 (Poperc)* | 0.164 (<.001) | *lHCPresubiculum (HC)* | 0.118 (0.001) | *TBV* | 0.551 (<.001) |
|  |  | *TBV* | 0.458 (<.001) | *lPFm (IPL)* | 0.172 (<.001) |  | |
|  |  |  |  | *r6d1 (SFS)* | 0.175 (<.001) |  |  |
|  |  |  |  | *lFo7 (Foperc)* | 0.203 (<.001) |  |  |
|  |  |  |  | *TBV* | 0.565 (<.001) |  |  |
|  |  |  |  | *lId2 (Insula)* | -0.081 (0.02) |  |  |
|  |  |  |  | *lTemporaltoParietal (GapMap)* | 0.09 (0.009) |  |  |
